# *In Vitro* Activation and Inhibition of Recombinant EGFR Tyrosine Kinase Expressed in *Escherichia coli*

**DOI:** 10.1155/2013/807284

**Published:** 2013-09-25

**Authors:** Jihene Elloumi-Mseddi, Karim Jellali, Sami Aifa

**Affiliations:** Centre of Biotechnology of Sfax, P.O. Box 1177, 3018 Sfax, Tunisia

## Abstract

The present work concerns the heterologous expression of the intracellular domain harbouring the tyrosine kinase activity of the epidermal growth factor receptor (EGFR). Protein expression was improved thanks to the deletion of a 13-amino acid peptide of the juxtamembrane region (JM). The recombinant proteins were produced as a glutathione S-transferase (GST) fusion in *Escherichia coli*, and the solubilisation was performed by sarkosyl addition during extraction. The produced proteins spontaneously dimerize allowing the activation of the tyrosine kinase domain in the presence of [*γ*-^32^P]ATP. The activity assay has revealed the autophosphorylation of EGFR proteins which was decreased in the presence of genistein. Our system could facilitate the screening of EGFR inhibitors without the need of adding an exogenous substrate.

## 1. Introduction

The epidermal growth factor receptor (EGFR) family consists of four members: EGFR/ErbB1/HER1, ErbB2/Neu/HER2, ErbB3/HER3, and ErbB4/HER4 which are transmembrane tyrosine kinases controlling cell proliferation, differentiation, and migration. Since they are involved in very important cell functions, any deregulation of signalling within the EGFR family could increase risks of tumor development facilitated by overexpression and/or mutation of the receptor in many solid tumours [1]. Therefore, understanding the structure/function relationship of the EGFR and related receptors has been the subject of many studies.

The EGFR is composed of an extracellular domain (ECD) harbouring the ligand binding site, a single transmembrane domain (amino acids 622–644), and a cytoplasmic domain. The latter hosts the tyrosine kinase (TK) catalytic domain (amino acids 683–958) which is flanked by a juxtamembrane (JM) domain (amino acids 645–682) and the C-terminal tail (amino acids 992 and 1186) containing the key tyrosine residues of autophosphorylation [2].

Moreover, EGFR and other members of its family are the most validated targets in the therapy of solid tumours. Indeed, there are three currently approved antibodies in clinical use: herceptin (an anti-HER2 approved to treat breast cancer overexpressing HER2), cetuximab, and panitumumab (anti-EGFR approved for treating metastatic colorectal cancer) [3]. Furthermore, there are three small-molecule inhibitors of the tyrosine kinase domain (TKIs) that interfere with the binding of ATP, also approved to treat non-small cell lung cancer (i.e., gefitinib, erlotinib, and lapatinib) [4]. These therapeutic molecules are limited by the development of resistance mutations such as T790M in EGFR, which necessitates the screening of new inhibitors. This screening is still costly, reflecting the need to seek an easier system to handle such as *E. coli*. The heterologous expression of EGFR-derived peptide sequences as fusion proteins in *E. coli* was reported more than twenty years ago, and the purified peptides were tested as substrates for the tyrosine kinase activities of the EGFR and c-src [5]. Nowadays, the recombinant active EGFR tyrosine kinase is still produced by the baculovirus system [6–8], which is more expensive compared to *E. coli*. The EGFR JM domain was also expressed for protein interaction studies such as calmodulin binding studies [9, 10]. Moreover, the intracellular EGFR domain or the ECD of some EGFR family members was also expressed in *E. coli* and thereafter purified in order to prepare antibodies [11, 12].

In this study, we have expressed recombinant intracellular EGFR domains fused to the GST tag in *E. coli*. It is known that the GST protein allowed protein dimerization and further could activate the tyrosine kinase [13], thus facilitating the screening of inhibitory compounds in this *in vitro* system.

## 2. Materials and Methods

### 2.1. Strains and Reagents

*E. coli* strain BL21 codon plus RIL (Stratagene) was used for GST-fusion protein expression, and JM109 competent bacteria (Promega) were used for plasmid construction and maintenance. Vector pLXSN, containing the cDNA of the full-length human EGF receptor [14], was a gift from Professor Axel Ullrich (Max-Planck-Institute, Martinsried, Germany). *E. coli* expression vector pGEX-6P-1 was purchased from Amersham Pharmacia Biotech. Anti-EGFR (sc-03) and anti-GST (sc-459) antibodies were obtained from Santa-Cruz Biotechnology. The horseradish peroxidase-conjugated secondary antibodies were purchased from Promega.

### 2.2. Plasmid Construction

The DNA fragment encoding the intracellular EGFR domain (TKJM) was amplified by PCR using Pfu-polymerase (Stratagene) and the pLXSN-HER plasmid as template. The following oligonucleotides were used, respectively, for PCR amplification of TKJM and its deleted form TKJMΔ [15] lacking the first 13 amino acids (TKJMΔ): 5′-CG GT CGA CT CAT GCG AAG GCG CCA CAT CG-3′ and 5′-CG GT CGA CTC ATG CTG CTG CAG GAG AGG GAG-3′ as forward primers, with SalI site (underlined) and 5′-GA GCG GCC GCC CCT CGT GGT TCA TGC TCC A-3′ as a reverse primer with NotI site (underlined). The obtained fragments were double-digested by SalI/NotI and inserted in pGEX6-P-1. We used S-300 columns (Amersham Pharmacia Biotech) to purify PCR products and the QIAquick PCR purification kit (Qiagen) to remove restriction enzymes from digested DNA before ligation. Ligation was performed by the “ready to go” T4 DNA ligase (Amersham Pharmacia Biotech). The resulting constructs, named pGEX-TKJM and pGEX-TKJMΔ, were verified by restriction enzymes and DNA sequencing.

### 2.3. Recombinant Protein Expression and Production

Single colonies of* E. coli* BL21 strains were grown overnight in 1 mL LB medium containing ampicillin (75 *μ*g/mL) at 37°C, 250 rpm. The cultures were then diluted to 1/50 in LB medium and incubated for 2 hours at 37°C, 250 rpm. Protein expression was induced by 0.1 mM IPTG for 3 hours at 37°C. Cells were harvested by centrifugation, and pellets were solubilised in SDS-PAGE loading buffer (Bio-Rad) and analysed in 10% minigels (Bio-Rad). Immunoblotting was performed with anti-EGFR or anti-GST as primary antibodies followed by horseradish-peroxidase-conjugated secondary antibodies. Detection was performed using the ECL chemiluminescence detection kit (GE Healthcare Bio-sciences). Alternatively, and in order to produce enough amounts of recombinant proteins, transformed BL21 strains were cultured in 100 mL M9 minimal medium and induced by 0.1 mM IPTG at 37°C, 250 rpm for 3 hours. The cell pellet was grounded with w/w aluminium oxide as described before [10]. The obtained powder was resuspended in the solubilising buffer 10 mM Tris-HCl pH 8, 150 mM NaCl, 1 mM EDTA, 5 mM dithiothreitol (DTT), 1 mM PMSF, and 1.5% sarkosyl (Sigma-Aldrich), and centrifuged at 12000 g for 5 min. Purification was performed after immobilisation on glutathione-Sepharose 4B beads, and elution was done as described by the manufacturer. 

### 2.4. Dimerization and Tyrosine Kinase Assays

BL21 strains of *E. coli* were cultured in M9 minimal medium and the recombinant proteins as described in the precedent section. The cell pellet was ground with w/w aluminium oxide as described before [10]. The obtained powder was resuspended in the tyrosine kinase buffer (25 mM Tris-HCl pH 7, 1 mM DTT, 5 mM MgCl_2_, and 1 mM EDTA) and centrifuged at 15000 g for 10 minutes. The recovered supernatant was mixed with glutathione-Sepharose 4B (GE Healthcare Bio-sciences) and incubated under gentle agitation in an end-over-end rotor at 4°C for 1 hour. After centrifugation, the Sepharose beads were resuspended in the tyrosine kinase buffer. [*γ*-^32^P]ATP (20 *μ*M, 4 *μ*Ci) was finally added, and the reaction was incubated for 15 min at 37°C. 100 *μ*M of Genistein was used for inhibiting the tyrosine kinase activity. The reaction was stopped by the SDS-PAGE loading buffer. After electrophoresis and western blotting, the phosphorylation was detected by the PhosphorImager (Bio-Rad) or autoradiography. Chemical cross-linking of recombinant proteins was performed as described by Fernandes et al. [16].

## 3. Results 

### 3.1. Expression of Recombinant EGFR Proteins in Fusion with GST

**Figure 1:**
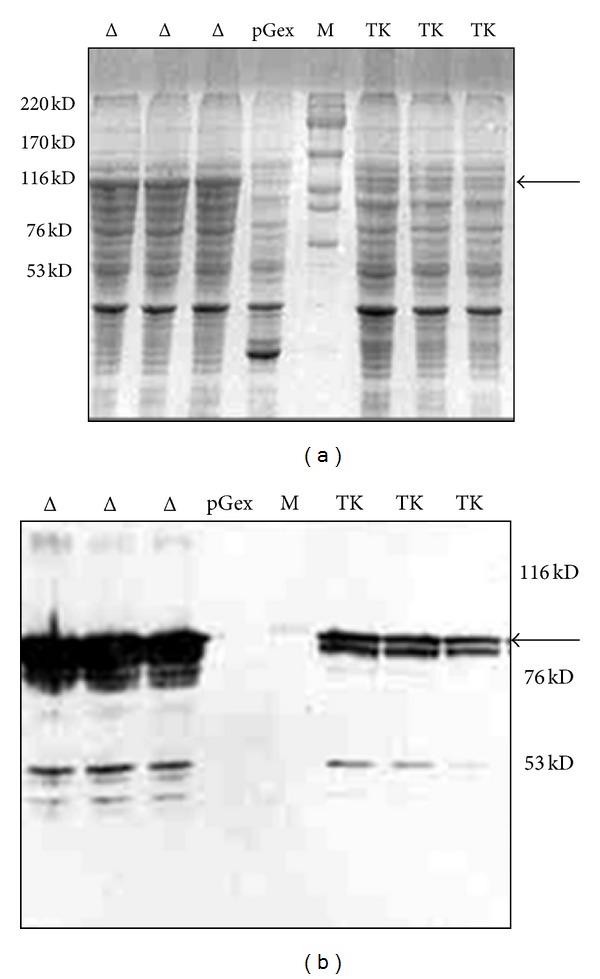
GST-TKJM and GST-TKJMΔ expression in *E. coli. E. coli* were transformed with the pGEX-TKJM (*TK*), pGEX-TKJMΔ (Δ), and the pGEX-6-P-1 (empty) plasmids, and the recombinant proteins are expressed as indicated in Section 2. The proteins were run in a 10% SDS-PAGE and stained with Coomassie blue (a) or subjected to western blot using an anti-GST antibody (b). Several *E. coli *clones expressing TKJM (TK) or TKJMΔ (Δ) are shown. The arrow points to the TKJM and TKJMΔ recombinant proteins.

After culture/induction of the recombinant strains for one hour with 0.1 mM of IPTG, cells were solubilised in loading buffer and total proteins were analyzed in SDS-PAGE. The analysis displays a better expression of GST-TKJMΔ compared to GST-TKJM (Figure 1(a)). This expression result was confirmed by western blot (Figure 1(b)). It seems that the deletion of the first 13-amino acids (TKJMΔ) improved the expression level. This 13 amino acid peptide of EGFR (R^645^–R^657^) is positively charged which could interfere with the protein heterologous expression and stabilisation. 

### 3.2. Dimerization and Activity Assays

**Figure 2:**
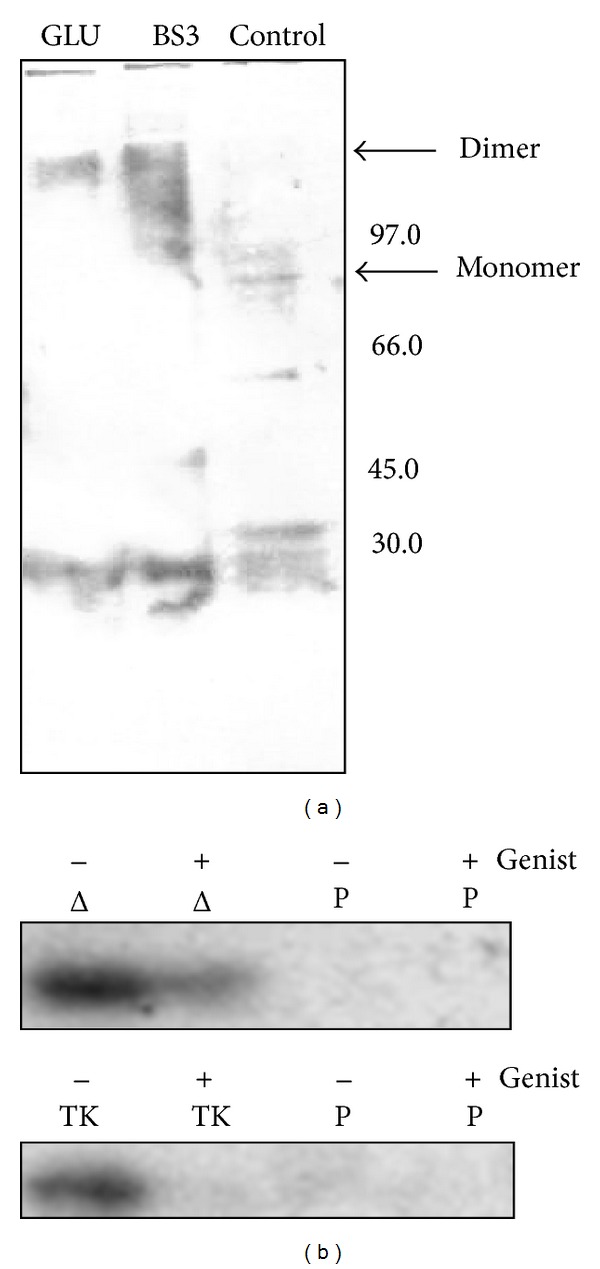
Dimerization and tyrosine kinase assays. Total protein extracts of *E. coli* transformed by pGEX-TKJMΔ (Δ) were treated with BS3 or glutaraldehyde (GLU) as indicated and analyzed by western blot with the anti-GST antibody (a). A control (C) without cross-linkage agent is also shown. The positions of the protein size marker (Bio-Rad) are indicated from 30 to 97 kD. The recombinant proteins were assayed with [*γ*-^32^P]ATP on immobilized glutathione-Sepharose beads as described in Section 2 (b). The empty pGEX-6P plasmid was used as control (P). Genistein (*Genist*) was added where indicated.

The EGFR activity depends on dimerization [17]. In our case, the EGFR proteins are fused to GST which could allow their dimerization since the GST protein tends to dimerize. To check the dimerization of our recombinant proteins, we used the cross-linking agents bissulfosuccinimidyl suberate (BS^3^) and glutaraldehyde. After treatment, the GST proteins are analysed by western blot with an anti-GST antibody. Chemical cross-linking by BS^3^ or glutaraldehyde showed that GST-fusion proteins preserve the ability of forming dimer (Figure 2(a)). Moreover, glutaraldehyde is showing better results of dimerization.

The recombinant proteins were assayed for tyrosine kinase activity of [*γ*-^32^P]ATP on the crude protein extracts or after immobilization to glutathione-Sepharose beads. The GST was used as negative control, and genistein was added to test the inhibition of the tyrosine kinase activity. 

Compared to GST, we noticed the incorporation of radioactivity in the EGFR intracellular proteins reflecting their transphosphorylation. This incorporation is more intense in the absence of genistein reflecting a specific tyrosine kinase activity of our recombinant proteins (Figure 2(b)); since GST-TKJMΔ is more expressed, genistein inhibition was not total. 

**Figure 3:**
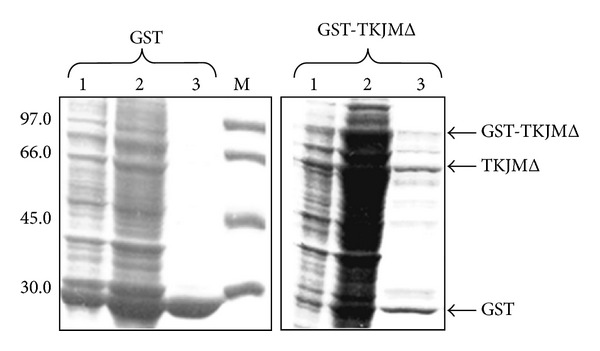
Recombinant protein purification. The recombinant GST-TKJMΔ and GST proteins were purified as described in Section 2. Lane 1: flow through; lane 2: ATP-wash; and lane 3: eluted proteins. The samples were run in 10% SDS/PAGE and stained with Coomassie blue. The arrow points to the TKJMΔ recombinant protein.

Therefore, we have performed the purification of the corresponding proteins as described in Section 2. The addition of sarkosyl during extraction has increased the solubilisation of the recombinant GST-TKJMΔ and facilitated the partial purification. GST cleaving is always occurring (Figure 3).

## 4. Discussion

We have succeeded with the expression of an active form of the intracellular domain of the EGFR harbouring the tyrosine kinase activity as GST-fusion proteins. The deletion of the basic amphiphilic 13-amino acid peptide of JM has allowed a better protein expression; this peptide was described as the calmodulin binding domain [9] and as the nuclear localisation signal of EGFR [18]. The use of enriched sarkosyl-soluble fractions of our proteins greatly facilitates the dimerization and activity assays. This allowed us to confirm the dimerization of GST in *E. coli* and our fusion proteins and the activation of the EGFR tyrosine kinase domain in *E. coli. *Our results confirmed the ability of GST to activate other proteins through dimerisation [19, 20].

Autophosphorylation of the intracellular EGFR segment was clearly shown, testifying the tyrosine kinase activity of the recombinant *E. coli* EGFR proteins. This tyrosine kinase activity detected is sensitive to inhibitors as is illustrated by using genistein. Thus, our study suggests the adoption of* E. coli* as a host expression of EGFR proteins fused to GST which could facilitate the screening of new antagonist molecules. Our results open the horizon for the development of more efficient inhibition tests for EGFR and in general for tyrosine kinase receptors via expression in *E. coli,* which might allow an easier selection of cancer antagonists targeting these receptors.

This strategy of GST-fusion proteins and inhibitor screening could be followed for any protein requiring dimerization for its activity.

## 5. Conclusions

EGFR is among the most targeted oncogenes in solid cancer by the use of monoclonal antibodies or small molecules inhibiting the tyrosine kinase activity (TKI). Screening for EGFR TKIs is based on the baculovirus recombinant protein that is still expensive.

The present work is showing the possibility to adopt the heterologous expression of EGFR tyrosine kinase in *E. coli* for screening TKIs. This test does not need protein purification which will further decrease the screening costs. Our strategy could be applied for other protein kinases that need inhibitors screening.
